# Beyond just a tight fortress: contribution of stroma to epithelial-mesenchymal transition in pancreatic cancer

**DOI:** 10.1038/s41392-020-00341-1

**Published:** 2020-10-30

**Authors:** Ashenafi Bulle, Kian-Huat Lim

**Affiliations:** Division of Oncology, Department of Internal Medicine, Barnes-Jewish Hospital and The Alvin J. Siteman Comprehensive Cancer Center, Washington University School of Medicine, St. Louis, MO 63110 USA

**Keywords:** Cancer microenvironment, Cell biology

## Abstract

Novel effective treatment is direly needed for patients with pancreatic ductal adenocarcinoma (PDAC). Therapeutics that target the driver mutations, especially the KRAS oncoprotein and its effector cascades, have been ineffective. It is increasing clear that the extensive fibro-inflammatory stroma (or desmoplasia) of PDAC plays an active role in the progression and therapeutic resistance of PDAC. The desmoplastic stroma is composed of dense extracellular matrix (ECM) deposited mainly by the cancer-associated-fibroblasts (CAFs) and infiltrated with various types of immune cells. The dense ECM functions as a physical barrier that limits tumor vasculatures and distribution of therapeutics to PDAC cells. In addition, mounting evidence have demonstrated that both CAFs and ECM promote PDAC cells aggressiveness through multiple mechanisms, particularly engagement of the epithelial-mesenchymal transition (EMT) program. Acquisition of a mesenchymal-like phenotype renders PDAC cells more invasive and resistant to therapy-induced apoptosis. Here, we critically review seminal and recent articles on the signaling mechanisms by which each stromal element promotes EMT in PDAC. We discussed the experimental models that are currently employed and best suited to study EMT in PDAC, which are instrumental in increasing the chance of successful clinical translation.

## Background

The aggressive nature of pancreatic ductal adenocarcinoma (PDAC) is driven intrinsically by powerful genetic mutations and extrinsically by a highly fibro-inflammatory (or desmoplastic) stroma, which typically constitutes up to 80–85% of the tumor bulk. The stroma consists of a dense acellular extracellular matrix (ECM) which is infiltrated with heterogeneous populations of cancer-associated-fibroblasts (CAFs), immune cells and endothelial cells.^1^ Work in the last two decades has now established that the PDAC stroma is not functionally inert, but instead actively shapes the behavior of PDAC cells and contribute to treatment resistance. The dense ECM creates a high-pressured barrier that collapses blood vessels, limiting the delivery and diffusion of oxygen, nutrients, and therapeutics to PDAC cells. The ensuing hypoxic and nutrient-poor tumor microenvironment (TME) also serves as a cradle for highly resilient PDAC cells that are metabolically adapted to this inhospitable environment.^2,3^ In addition, CAFs secrete various chemokines and cytokines that enhance tumor progression and therapeutic resistance. A critical pathophysiological process that associates PDAC cells with these feats is epithelial-mesenchymal transition (EMT). During the process of EMT, cancer cells loss epithelial markers such as E-cadherin, certain cytokeratins, occludin, and claudin, and gain mesenchymal markers such as vimentin, N-cadherin and fibronectin. These changes result in disruption of normal cell–cell adhesion, loss of cellular polarity, remodeling of the cytoskeleton, and alteration in cell–matrix adhesion, which collectively translate into enhanced migratory, invasive and metastatic properties.^4–7^ PDAC cells that assume the more mesenchymal phenotypes are also more resistant to cytotoxic or cell-cycle disrupting therapeutics such as chemotherapy and MAPK inhibitors, partly explaining why these treatments are neither very effective nor durable in the clinic. Recent evidence also show that EMT is associated with immune evasion and potentially resistance to immunotherapy.^8^ Therefore, targeting EMT represents a solid therapeutic strategy. Several excellent reviews were published in recent years on the underlying signaling mechanisms and role of EMT in cancer,^5–7,9–12^ as well as the pathogenic role of stroma in PDAC.^2,3,13^ However, a dedicated review on how stroma promotes EMT in PDAC is lacking. In this study we critically reviewed seminal and recent literature and provided a focused review on signaling mechanisms by which distinct elements of stroma, namely CAFs, ECM and hypoxia, promote EMT of PDAC cells. We shed light on these complicated and interlocking mechanisms that collectively drive EMT in PDAC.

## Epithelial-mesenchymal transition (EMT)

EMT is a dynamic, reversible process through which epithelial cells assume a mesenchymal-like phenotype, defined by changes in cell morphology, acquisition of mesenchymal markers and migratory function. The EMT process is orchestrated by a suite of transcriptions factors (EMT-TFs) including Snai1 (Snail), Snai2 (Slug), Zeb1, Zeb2, and Twist.^12,14^ These TFs also happen to be actively utilized by stem cells and progenitor cells during embryonic development, but has later be shown to occur very commonly in human cancers including PDAC.^15,16^ Instead of full EMT, most cancer cells undergo various degrees and different phenotypic versions of “partial” EMT, usually in response to environmental clues, to adapt and survive. By re-expressing these stem cell markers, partial-EMT cells are sometimes dubbed as being more “stem-like” in properties, which include less proliferative and more migratory capabilities. Acquisition of EMT also alters the intrinsic cell death machineries that translate into therapeutic resistance. Various signaling pathways are involved in expression of the EMT-TFs, and these include the TGF-β, Notch, Wnt/β-catenin and inflammatory JAK/STAT and NF-κb cascades.^11^ In a genetically engineered mouse model (GEMM, *PDX-Cre;p53*^*flox/WT*^*;LSL-KRAS*^*G12D*^, aka KPC mice) of PDAC, lineage-tracing experiments showed that EMT occurs at very early stage of neoplastic progression way before emergence of frank PDAC tumors, precipitated predominantly by inflammation.^17^

Specifically, chronic Inflammation promotes tumor progression by changing the tumor microenvironment at the primary site of neoplasia and promoting tumor invasion and dissemination.^17,18^ As the low grade pre-invasive lesion advanced to chronic inflammation, various protumoral cellular and acellular (fibrotic tissue) factors are recruited in to the TME (details in the next sections). The cellular components secrete various cytokines and growth factors, whereas the acellular fibrotic tissue increases intertumoral pressure and formation of structurally and functionally abnormal blood vessels, resulting in severe hypoxia.^19,20^ These changes promote EMT that endow the tumor cells with enhanced invasiveness and metastatic capability.^4,21^ Importantly, studies of human PDAC samples showed no statistical difference in the level of desmoplasia between the primary and metastatic lesions.^22^

Analyses of GEMMs and human patients showed that PDAC cells that have undergone EMT and extravasated to the circulation retain EMT markers including vimentin and Zeb1.^17,23^ In addition, studies in GEMMs showed that circulating PDAC cells that exhibit EMT markers have stem-like properties, which allow them to initiate tumor formation in distant sites.^17^ These studies show that a subset of PDAC cells may retain an EMT or stem-like feature even in the apparent absence of CAFs, at least for a short period of time during distant metastasis. Importantly, EMT is reversible as cancer cells retain the ability to revert to epithelial phenotype (MET, or mesenchymal-epithelial transition) upon disengagement of these environmental clues. Therefore, EMT is not entirely a binary process, but instead is dynamic, pliable, and subject to fine-tuning by the environmental clues.

## Components of PDAC tumor microenvironment (TME) that drive EMT

**Fig. 1:**
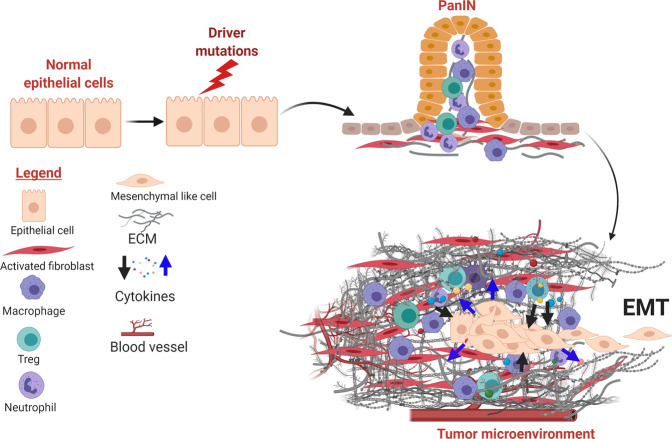
PDAC initiation and establishment of desmoplastic stroma. The desmoplastic stroma typically precedes and co-evolves with neoplastic progression in PDAC. The stroma is characterized by diverse cellular and non-cellular components that undergo constant remodeling in response to tumor progression and therapeutic intervention. In addition to intrinsic genetic mutations, a subset of PDAC cells engage the EMT program under stromal clues and metastasize

The desmoplastic stroma of PDAC can arise from the pre-existing chronic inflammation that predispose and accompany neoplastic progression,^1,24^ as well as cancer-associated inflammation (Fig. 1). Patients with chronic pancreatitis are at increased risks for developing PDAC.^25^ In PDAC GEMMs, chemical-induced acute or chronic pancreatitis greatly accelerates the pace and enhances the incidence of PDAC development.^25–28^ Conversely, precancerous or neoplastic cells can themselves serve as inflammatory triggers to the host immune system, stirring innate and adaptive inflammatory responses that further exacerbates the stromal reaction.^26–30^ CAFs are the major producers of ECM proteins, which consist predominantly of collagens, fibronectin, and laminin,^31^ although recent evidence suggests PDAC cells can also contribute to deposition of the ECM.^32^ Chronic, high-ordered structuring of these ECM proteins results in a stiff, three-dimensional mesh which, in combination with high molecular weight glycosaminoglycans such as hyaluronan, form a milieu with high interstitial pressure that collapses blood vessels and limits delivery of oxygen and nutrients.^33,34^ In addition to these biophysical properties, ECM molecules have signaling function by triggering membrane receptors on PDAC cells and engaging pathways that contribute to EMT.^31^ In addition, CAFs secrete several humoral factors such as TGF-β, IL-1α/β, IL-6, CXCL12, FGF, EGF, TNF-α,^35–39^ which promote survival, proliferation and EMT of PDAC cells.^39–43^

### Cancer-associated-fibroblasts (CAFs)

Accurate identification of CAFs has been a challenge in the field. Although markers such as alpha-smooth muscle actin (α-SMA), fibroblast-specific protein 1 (FSP1), fibroblast activation protein (FAP), and platelet-derived growth factor receptors alpha/beta (PDGFRα/β), are commonly used to identify CAFs, these markers are also be present in epithelial cells (not specific) and not universally present in all CAFs populations (not sensitive). The recent consensus from the Barbury Center Meeting held by experts in the field recommended that “cells negative for epithelial, endothelial and leukocyte markers with elongated morphology and lacking the mutations found within cancer cells might be considered CAFs”.^44^ Furthermore, the consensus recommended that categorization of CAFs should be determined mainly by function, informed by direct experimental evidence and in some cancers, clinical correlations.^44^ Therefore, CAFs should be studied in experimental conditions that maximally mimic the actual TME, and also standardized to improve reproducibility.

### Origins and subtypes of PDAC CAFs

CAFs are diverse in origin and heterogenous in subtypes and function.^45^ Obtaining longitudinal human pancreas samples from pre-malignant to malignant stages is practically impossible and even if available, are typically limited in material and cross-sectional in nature. Studies to define the origins of CAFs in PDAC have mostly been done in GEMMs, but progress is stifled by a lack of truly CAF-specific markers to enable accurate lineage tracing. Evidence from the constitutive or inducible KPC (*Pdx-Cre/p53*^*mut/wt*^*/LSL-KRAS*^*G12D*^) GEMMs showed that stromal fibroblasts, defined by positive α-SMA staining, around the microscopic precancerous lesions called pancreatic intraepithelial neoplasia (PanIN), undergo expansion even before invasive PDAC clusters appear,^46,47^ suggesting that the bulk of CAFs probably originate from local precursor fibroblasts or pancreatic stellate cells,^48^ at least in early stage of PDAC development. However, additional origins of CAFs have been proposed in other cancer types, and these include bone-marrow-derived mesenchymal stem cells (MSCs), or trans-differentiation from adipocytes,^49,50^ pericytes,^51^ smooth muscle cells, and endothelial cells.^52^ However, direct evidence to support derivation of PDAC CAFs from these origins is lacking.

Besides producing ECM, CAFs actively engage in reciprocal signaling exchange with PDAC and the infiltrative immune cells during tumor progression. For decades, most studies have shown CAFs to be protumorigenic, based largely on co-culture and co-injection experiments.^53,54^ However, near global depletion of stromal CAFs in GEMMs paradoxically accelerates PDAC progression by inducing immune-suppression, suggesting that CAFs have both tumor-promoting and -restraining roles.^55–58^ The recent advent of powerful single-cell techniques now revealed the existence of different transcriptomic subtypes of CAFs, each with distinct functions.^59,60^ Such functional diversification of CAFs appeared to be related to their distance from PDAC cells and residing niche, and interestingly are interchangeable. Through careful analyses of α-SMA and FAP expression in PDAC tumor sections, Öhlund et al showed that dual α-SMA^+^ and FAP^+^ CAFs (called myofibrobasts or myCAFs) are in direct proximity to PDAC cells. On the other hand, FAP^+^ CAFs that are located distant away from PDAC cells stain weakly for α-SMA but instead express high levels of inflammatory cytokines including IL-6, IL-1, and LIF, and these are called inflammatory CAFs (iCAFs).^59^ It was proposed that myCAFs are possibly tumor restrictive, whereas iCAFs promotes tumor progression, partly explaining the observation that depletion of α-SMA^+^ CAFs accelerates PDAC progression in GEMMs.^61^ Importantly, these two phenotypes are interchangeable, based on spatial and biochemical niche of culture conditions. For instance, IL-1 was shown to induce conversion to iCAF by through the JAK-STAT pathway; whereas TGF-β downregulates expression of IL-1 receptor 1 expression (IL-1R1) and promotes conversion to myCAFs.^62^ Therefore, JAK inhibition may be useful in blocking conversion to iCAFs, which have protumorigenic properties. However, addition of JAK1/2 inhibitor (ruxolitinib) to capecitabine failed to improve overall survival in patients with metastatic PDAC,^63^ highlighting the need to explore other targeted approach to durably maintain the myCAF phenotype and curb the IL-1R pathway. For the latter, targeting IRAK4, the master kinase downstream of IL-1R signaling, represents a promising approach. Silencing of IRAK4 in CAFs dramatically reduce the ability of CAFs to secrete inflammatory cytokines and PDAC growth in vivo.^54^ Interestingly, using single-cell RNA sequencing in mouse PDAC tumors, a third distinct CAF subtype was identified and termed antigen- presenting CAFs (apCAFs). The apCAFs are also present in human PDAC albeit in much smaller abundance. The apCAFs have increased expression of MHC class II and therefore may be able to present antigens to CD4^+^ T cells. However, lack of co-stimulatory molecules on apCAFs may result in incomplete CD4^+^ T cell activation. Therefore apCAFs are proposed to blunt anti-tumor response by outcompeting the scarce dendritic cells for access to CD4^+^ T cells.^60^ Signaling pathway leading to acquisition of apCAF phenotype, and the strategies to confirm the role of apCAFs which is necessary for therapeutic intervention are still unclear.

### Contributions of CAFs to EMT

The histological progression of PDAC is driven collectively by PDAC cells and cell types that they subvert, particularly CAFs. Because CAFs are not oncogenically mutated, their biological outputs are passively controlled by adjacent PDAC cells, although additional epigenetic modification may enable them to behave more autonomously.^64^ However, once subverted, CAFs can in turn promotes the aggressiveness, including EMT, of PDAC cells.

### Secreted factors from CAFs drive PDAC EMT

**Fig 2:**
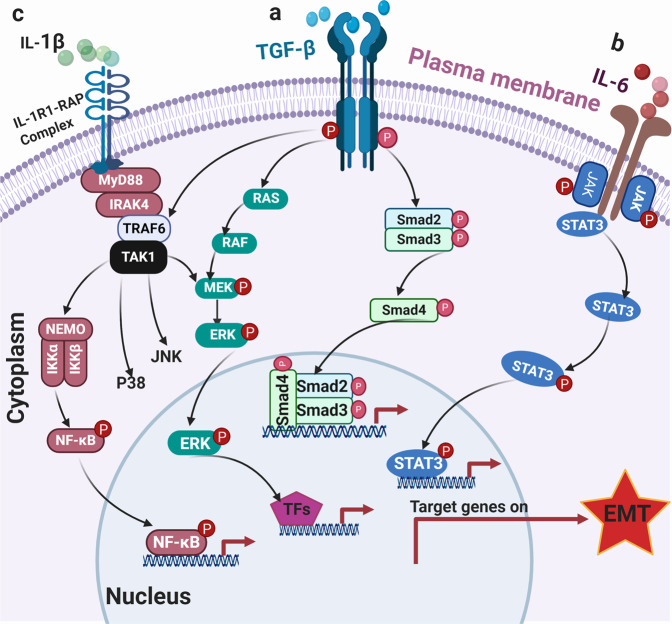
Collective contribution of CAFs and immune cells to EMT. CAFs interact with both the tumor cells and immune cells especially M2 TAMs through secreted cytokines and chemokines, resulting in PDAC EMT

The PDAC TME is rife with various cytokines and chemokines produced by CAFs, PDAC, and immune cells, and many of which potent inducers of PDAC EMT (Fig. 2). Here we review the signaling mechanism by which the best-described cytokines, TGF-β, IL-1, IL-6, and TNFα, induce EMT.^65^

#### TGF-β

**Fig 3:**
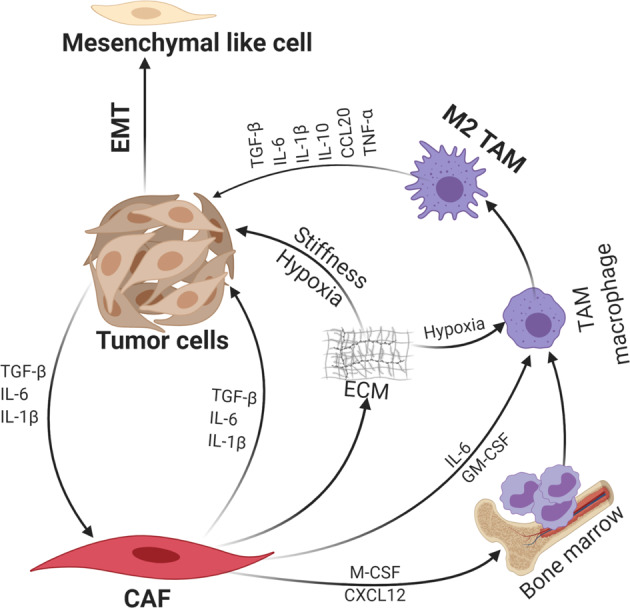
Role of TGF-β, IL-6, and IL-1β signaling pathways in PDAC EMT. **a** TGF-β signaling pathway can be SMAD-dependent or independent. In SMAD-dependent setting, TGF-β binds its receptor complex (TGF-β receptors I/II dimer) and phosphorylate SMAD proteins and subsequently transported into the nucleus to induce EMT-TFs. Activation of TGFR can also engage MAPK and NF-kB signaling to transactivate EMT-TFs. **b** Engagement of the IL-6R activates JAK kinases which phospho-activate STAT3 transcription factor, leading to upregulation of EMT-TFs. **c** Activation of IL-1R engages IRAK4-TAK1 and the downstream NF-κB, JNK, and p38 MAPK cascades to promote EMT

Transforming growth factor-β (TGF-β) is a pleotropic cytokine, which controls several cellular functions including EMT, proliferation, and survival. In many cancer types including PDAC, TGF-β is the major inducer of EMT.^66^ Engagement of the TGF-β receptor complex (TGF-βRI/II) leads to phosphorylation of SMAD2 and SMAD3 transcription factors, which heterotrimerize with SMAD4 and translocate into the nucleus to activate or repress target genes^67,68^ (Fig. 3). In normal cells, TGF-β pathway activation leads to cell-cycle arrest through SMAD-dependent downregulation of c-Myc leading to upregulated transcription of CDK inhibitors p15 and p27, as well as abrogation of prereplication complex, which causes G1/S arrest.^69^ Simultaneously, activated SMAD2/3/4 drive transcription of several EMT-TFs including Snai1, Snai2, Zeb1, Zeb2, and TWIST.^70^ This results in release of EMT cells from the confine of adjacent epithelial cells and basement membrane, which can then migrate to distant organs to differentiate and repopulate. Importantly, TGF-β signaling is quenched by negative feedback mechanisms. For instance, upregulation of inhibitory SMADs (SMAD6 and SMAD7) blocks phosphorylation of SMAD3/4 and recruits E3 ligases Smurf1/2 to degrade TGF-β receptors.^71^ Therefore, tightly regulated TGF-β signaling ensures normal embryonic development, wound healing process and tissue regeneration. In addition to the SMADs, TGF-β ligation also leads to relatively rapid activation of RAS, ERK, TAK1, JNK, p38 MAPK, and the IKK-NF-κB pathways, leading to transcription of EMT-TFs and also TGF-β, providing another mechanism that amplifies TGF-β response.^72,73^ However, the cellular context and exact mechanism by which these pathways tune EMT remains poorly characterized in PDAC.

In PDAC, the impact of TGF-β signaling is highly dependent on cellular context and concurrent genetic alterations.^74^ While on one hand tumor-intrinsic TGF-β signaling is growth suppressive, stromal TGF-β signaling leads to fibrosis and immune escape. In PDAC cells with intact SMAD signaling, TGF-β activation results in engagement of EMT, which is accompanied by reduced proliferation and apoptosis.^75,76^ In support, pancreas-specific mono- or biallelic deletion of *TGFRII*, which encodes the primary receptor for TGF-β, cooperates with oncogenic *KRAS*^*G12D*^ in accelerating development of aggressive PDAC in GEMMs,^77^ demonstrating that tumor-intrinsic TGF-β signaling is tumor-suppressive. However, PDAC cells that eventually escaped the suppressive effect of TGF-β have re-expressed *ID1*. *ID1* is a pancreas progenitor gene, which encodes a helix-loop-helix (HLH) protein that heterodimerizes with bHLH transcription factors, and re-expression of *ID1* protects PDAC cells from TGF-β-induced apoptosis.^78^ In 60% of human PDAC, *SMAD4* is silenced by allelic deletion, intragenic mutation or epigenetic silencing, which correlate with widespread metastasis.^79^ In PDAC GEMMs, pancreas-specific deletion of *SMAD4* cooperates with *KRAS*^*G12D*^ to accelerate development of IPMN, a precancerous lesion, but progression to invasive PDAC occurs at relatively low penetrance (2/12 mice).^80,81^ Importantly, when further crossed with *INK4a/Arf*^*flox/flox*^ mice, *SMAD4-null KRAS*^*G12D*^ PDAC tumors developed at higher frequency but displayed significantly more prominent epithelial identity including higher E-cadherin and lower Slug expression, upholding a role of *SMAD4* in promoting EMT.^80^ A recent report showed that *SMAD4-null* PDAC cells survive TGF-β-induces apoptosis through expression stem cell factor Sox4, which cooperates with Klf5, a lineage survival factor, to restore tumor-initiating ability.^76^

These genetic models clearly demonstrate that tumor-intrinsic TGF-β signaling is growth-prohibitive. However, the global impact of TGF-β signaling is protumorigenic. In GEMM, global deletion of *TGFRII* impedes PDAC development via inhibition of stromal fibrosis, and restoration of anti-tumor immune function,^75^ indicating that stromal TGF-β signaling is protumorigenic. In support, nearly half of PDAC tumors showed enhanced TGF-β or TGFRII expression by immunohistochemistry, which correlate with poor survival,^82,83^ overall upholding the rationale for moving systemic inhibition of PDAC in clinical trials, although the correct combinations remain to be determined.

#### Interleukin-6

Interleukin-6 (IL-6) is a major inflammatory cytokine that is required for *RAS*-induced tumorigenesis.^84^ In addition, secretion of IL-6 by pancreatic stellate cells (PSCs) and myeloid cells activates STAT3 signaling to promote progression from PanIN to PDAC.^85,86^ Ligation of IL-6R results in phosphorylation of JAK kinases which phospho-activate the STAT3 transcription factor. Deletion of *STAT3* significantly lowered spontaneous and caerulein-induced progression of PanIN to PDAC in GEMMs.^87,88^ Furthermore, lineage-tracing model showed that EMT precedes frank tumor progression in GEMMs, which is greatly accelerated by cerulean-induced inflammation.^17^ By inference, loss of *STAT3* may retard inflammation- induced EMT, but this remains to be determined. Notably, the role of STAT3 in EMT is complex. CRISPR/Cas9 ablation of *STAT3* in murine *KRAS*^*G12D*^*/p53*-null cells results in formation of tumor xenografts that are anaplastic in histology and displayed EMT characteristics including loss of keratin, E-cadherin and acquisition of SMA and ACTA2.^89^ Mechanism leading to changes of these markers is unclear. Remarkably, forced expression of phosphomimetic STAT3 S727E mutant, but not wild-type or the hyperactive Y640F mutants, induces partial-EMT phenotype evidenced by downregulation of EpCAM, CD133 and a mixed epithelial-mesenchymal histology.^89^ Mechanistically, activated STAT3 enhances *vimentin* gene expression by binding to the antisilencer element upstream of *vimentin* promoter, where it binds and overcome the repressor function of ZBP-89 and allowing *vimentin* to be transcribed.^90^ Interestingly, PDAC cells that have undergone EMT upregulates IL-6 transcription via TWIST to sustain tumor inflammation,^91^ and perhaps further sustain EMT via autocrine JAK-STAT3 activation. Therefore, the contribution of STAT3 to EMT is likely context dependent: pro-EMT in the setting of inflammation as in the presence of IL-6, but sustained loss of *STAT3* likely results in adaptive outgrowth of EMT population through mechanisms that are largely unclear.

#### IL-1α/β and TNF-α

Constitutive activation of the NF-κB transcription factors is present in two third of PDAC and is tightly associated with tumor fibrosis, chemoresistance and poor prognosis in PDAC.^92^ One of the many important biological outputs from NF-κB activation is induction of inflammation, a hallmark of the PDAC TME and an important driving mechanism of EMT. In fact, activation of the NF-κB cascade is required for *KRAS*-induced PDAC development in GEMMs.^93^ Activation of the canonical NF-κB cascade is driven by IRAK4-TAK1-IKK axis following engagement of the IL-1R by autocrine and paracrine IL-1α and IL-1β secretion, resulting in increased chemoresistance, invasion, and metastasis.^54,92,94^ TGF-β-induced EMT is also partly blocked by overexpression of dominant negative ikbα mutant, demonstrating the contribution of the canonical NF-κB cascade in TGF-β-induced EMT.^95^ Expression of TNF-α or activated IKK mutant induces EMT through upregulation of vimentin, ZEB1, and downregulation of E-cadherin.^95^ In addition, TNF-α activates the NF-κB pathway, leading to induction of COP9 signalosome 2 (CSN2), which blocks ubiquitination and proteasomal degradation of Snail to promote EMT.^96^ The NF-κB cascade can also be activated by overexpression of IGFBP2, which degrades PTEN and activates the PI3K-AKT-IKK axis, leading to downregulation of E-cadherin, upregulation of vimentin and increased migration and invasion of PDAC cells.^97^ Therefore, targeting the NF-kB cascade at the convergent nodes from these upstream receptors, particularly TAK1 and IKK kinases, are promising therapeutic strategies, although effective, specific, and safe TAK1 and IKK inhibitors are still not available.

### Contributions of ECM to EMT

The dense desmoplastic stroma of PDAC contains a large amount of EMC that includes the fibrillar collagen, fibronectin, laminin, as well as proteoglycans such as hyaluronic acid (HA, or hyaluronan). The building blocks for these ECM proteins are produced mainly by CAFs, and to a much smaller degree, cancer cells. Besides functioning as physical barrier, these elements have signaling function that contributes to EMT of PDAC cells.

#### Collagens

Mass spectrophotometric studies of PDAC from patient samples and GEMMs showed that collagens are the most abundant, comprising 90% of ECM in PDAC. Of all the different types of collagen, type I and III are the most prominent, whose abundance increases during progression from PanIN to PDAC.^32^ The less abundant type IV collagen is the main component of basement membrane which, along with laminin, separates PDAC and endothelial cells from the remaining stroma.

Dense stromal deposition of type I and III collagens results in a stiff TME that not only restricts neovascularization, but also shapes the behavior of PDAC cells via various mechanotransduction pathways.^98^ The integrins and discoidin domain receptor (DDR) are two major families of cellular receptors that sense collagen in the TME. The integrins are heterodimeric transmembrane receptors that are formed by noncovalent association between 18 different α and 8 different β subunits, resulting in 24 members, each with distinct ligand (collagen, fibronectin and laminins) recognition.^99^ Ligation with the ECM proteins results in clustering of integrins, forming focal adhesions on the cell membrane where the integrins utilize linkers such as vinculin and paxillin to connect with intracellular actin cytoskeleton.^100^ Such ECM-actin interaction results in activation of signaling pathways including the FAK/SFK, Fyn/YES, and Rac/Cdc42 cascades that initiate EMT to facilitate cellular migration.^101^ Specifically, FAK activates p130CAS/paxillin, which in turns engages Rho GTPases to form focal adhesions necessary for actin polymerization. Activated paxillin also activates JNK kinase, which phosphorylates c-Jun transcription factor and upregulation of N-cadherin.^102,103^ Another mechanism by which FAK promotes EMT is via activation of Yes-associated protein (YAP) and its homologous protein Transcriptional Co-Activator With PDZ-Binding Motif (TAZ). Activated YAP-TAZ translocate to the nucleus and interact with the TEA domain family members (TEAD) to transactivate genes that drive EMT, which include *RHOA, CDC42, RAC, SLUG, SNAIL*, and *ZEB*.^104–106^ Notably, formation of focal adhesions is proportionate to the external mechanical force,^107^ and thus in the stiff PDAC TME these adhesion pathways are most likely constantly engaged. In support, the epithelia of human and murine PDAC exhibited strong activation of FAK by phosphorylated-FAK staining, whereas this is almost absent in normal epithelia. Pharmacologic suppression of FAK reduces collagen deposition in KPC GEMM and potentiates response to immunotherapy and chemotherapy.^108^

Besides the integrins, the discoidin domain receptors (DDR), which consists of DDR1 and DDR2, are a family of receptor tyrosine kinase that sense collagens. Stimulation of DDR1 with collagens were reported to activate several signaling cascades including PI3K/Akt and Ras/ERK MAPK pathways.^109^ In PDAC, DDR1 and its downstream protein tyrosine kinase 2 (Pyk2) are required, in addition to integrin signaling, in collagen-induced N-cadherin switch.^102^ Inhibition of DDR1 reduces collagen-induced PDAC tumorigenicity via suppression of protein tyrosine kinase 2 (Pyk2) and pseudopodium-enriched atypical kinase 1 (PEAK1) activation, thereby enhancing the effect of chemotherapy in preclinical models.^110^

#### Fibronectin

Fibronectin (FN) is a high molecular weight glycoprotein composed of repeats of different type of FN isoforms generated from alternative splicing of the fibronectin pre-mRNA.^111^ Fibronectin binds predominantly the integrins, especially integrin α5β1, to activate FAK and Rho GTPases to promote EMT in various solid tumors.^112^ Although direct experimental evidence linking fibronectin and EMT is lacking in PDAC, similar mechanisms are expected to be involved. In addition, fibronectin binds collagen and is expected to positively contribute to collagen signaling and hence EMT in PDAC.

#### Hyaluronan

Investigation of HA as a therapeutic target in PDAC biology has been of intense interest in recent years. Hyaluronan is a high molecular weight linear glycosaminoglycan (GAG) that consists of repeating N-acetyl glucosamine and glucuronic acid units assembled by the cytoplasmic HA synthases, HAS1, HAS2, and HAS3.^113^ The growing HA polymers are extruded through the plasma membrane into the extracellular space where they are incorporated into the pre-existing ECM. On the other hand, HA is degraded by hyaluronidases (HYAL1-4, HYALP1, and PH20). Of these, HYAL1 and HYAL2 are widely distributed throughout tissues and probably the main enzymes that degrade HA.^113,114^ The accumulated HA matrix serves as a water-absorbent that maintains tissue hydration and homeostasis. However, excessive HA deposition, as commonly found in PDAC tumors, results in high interstitial pressure that collapses tumor vasculature and limits delivery of therapeutics.^115^ In support, high HA and HAS2 expression, and low HYAL1 expression were independent factors associated with poor postoperative survival in PDAC patients.^116^ In PDAC mouse model, addition of hyaluronidase reduces intratumoral hyaluronan content and interstitial pressure, which allows re-expansion of the microvasculature and increases delivery of chemotherapy into PDAC TME, thereby lengthening survival of treated mice.^117^ Unfortunately, addition of pegylated PH20 (PEGPH20) to chemotherapy FOLFIRINOX or gemctabine/nab-paclitaxel failed to improve outcome of PDAC patients and will be discussed in later section.

Besides its biophysical functions, HA instructs behavior of PDAC cells through interactions with their surface receptors, especially CD44 and receptor for HA‐mediated motility (RHAMM).^118^ Importantly, CD44 is one of the putative stem cell markers in PDAC,^119^ signifying its supportive role in EMT. Furthermore, decades of studies showed that CD44 participates in almost every aspects of malignant phenotypes of cancer cells,^120^ largely enabled by its multifarious signaling capabilities. Besides the standard-length isoform (CD44s), CD44 is alternatively spliced into variants (CD44v1-10), which subsequently undergo modifications by N‐ and O‐linked glycosylation that affects their substrate binding affinity and specificity, and hence downstream signaling pathways. Of all variants of CD44, CD44v6 has been shown to promote tumor progression and metastatic spread in many solid tumors.^121^ CD44 can also undergo stepwise proteolytic cleavage by type 1 matrix metalloprotease and γ-secretase, releasing a 12kDa intracellular domain (ICD) that can translocate to the nucleus to transactivate CD44, as well as genes that promote survival, oxidative glycolysis, invasion, and stemness factors including *NANOG, SOX2, OCT4 c-MYC*, and *TWIST1*.^122–125^ Upon HA binding, both CD44 and RHAMM function as a co‐receptors to activate other transmembrane tyrosine kinases, including epidermal growth factor receptor, c‐MET, and platelet‐derived growth factor receptor to promote tumor progression and therapeutic resistance.^117,126,127^ In addition, CD44 and RHAMM ligation also activates kinases including ERK, FAK, Src to promote cell migration, invasion, and survival.^118,128–130^ Importantly, downstream signaling and functional output of CD44 ligation is greatly dependent on the molecular weight of bound HA.^131^ During inflammation and in cancer stroma, the turnover of HA is enhanced, yielding richer species of medium and low molecular weight HA. CD44 that binds to these species of HA are more inclined to undergo proteolytic cleavage to yield CD44 ICD, which is pro-EMT.^132,133^ Therefore, the HA-CD44 interaction is clearly a major mechanism that drives EMT in PDAC, making it an attractive therapeutic target.

### Contributions of hypoxia to EMT

The combination of low perfusion, defective tumor vasculature and enhanced intercellular competition for oxygen creates an extremely hypoxic TME. Chronic hypoxia plays an active role in promoting cancer aggressiveness and treatment resistance, in part through promoting EMT.^134^ Adaptation to hypoxia is driven mainly by hypoxia inducible factors (HIFs).^135^ HIFs are heterodimers consisting of an oxygen-sensitive α subunit (HIF-1α, HIF-2α, and HIF-3α) and a constitutively expressed β subunit. In normoxia, HIF-1α is hydroxylated, which enables it to bind and be polyubiquitinated by the von Hippel-Lindau (VHL) E3 ligase and targeted for proteasomal degradation. Hypoxia results in loss of hydroxylation and subsequently stabilization and accumulation of HIF-1α, which enters the nucleus, dimerizes with HIF-1β and transactivates target genes. Notably, HIF-1α has been shown to bind the hypoxia-responsive elements (HREs) in the promoter region of EMT-TF genes including *TWIST1, Snail, Slug*, and *ZEB1* to drive EMT^.^^134,136^ Interestingly, co-expression of HIF-1α and Slug to mimic hypoxia more readily induces EMT signaling and migratory potential in PDAC population that are more stem-like, as determined by expression levels of ALDH, E-cadherin, and vimentin,^137^ indicating that hypoxia may cooperate with other environmental and cell-intrinsic processes to intensify EMT. Another mechanism by which hypoxia may promote EMT is via sustaining NOTCH signaling. In this setting, hypoxia stabilizes Notch 1 intracellular domain (ICD), which recruits HIF-1α and binds to Notch-responsive promoters to transactivate Notch target genes including *HEY-2* and *PGK1* to maintain stem cell state.^138^

### Contributions of immune cells to EMT

The PDAC TME is rife with various kinds of immune cells, particularly myeloid cells, which play an active role in maintaining desmoplasia and stifle anti-tumor response. Profound numbers of tumor-associated macrophages (TAMs) accumulate during PDAC progression.^1^ TAMs can originate from embryonic development, which is the main source of pancreas-resident macrophages, and expand through in situ proliferation where they exhibit a profibrotic transcriptional profile, indicative of their roles in cancer-related inflammation, immune escape, matrix remodeling, and metastasis.^139,140^ Monocyte-derived TAMs play potent roles in antigen presentation and can differentiate into antitumorigenic M1- or (protumorigenic) M2-phenotypes. Notably, CAFs promote an immunosuppressive microenvironment through induction and accumulation of M2-polarized TAMs.^141^ CAF-derived chemokine (C-X-C motif) ligand 12 (CXCL12)^142–144^ and macrophage colony-stimulating factor (M-CSF)^40,145^ are well reported factors that effectively recruit monocytes to the tumor tissue. Furthermore, CAF-derived IL-6 and GM-CSF cooperate to induce trans-differentiation of tumor-resident macrophages to M2 macrophages.^146–148^ Therefore, the iCAFs are more likely the subpopulation that induces M2 polarization, although this will need to be verified by direct experimental evidence. In addition, the hypoxic tumor microenvironment can also triggers M2-polarized TAMs.^149–151^ Once polarized, M2 TAMs secrete various growth factors and cytokines, including TGF-β,^152,153^ interleukin-IL-10, IL-6, TNF-α,^40,154,155^ IL-1β,^156,157^ migration inhibitory factor (MIF)^158^, and chemokines such as CCL20,^159^ which are all capable of promoting EMT as previously described. In addition, gemcitabine treatment results in robust infiltration of M2-polarized TAMs which secrete TNFα, TGF-β, and IL-6, leading to EMT of PDAC cells.^160^ Therefore, tumor infiltrative M2 TAMs are another major propeller of PDAC EMT via providing various cytokines and chemokines.

## Preclinical models to study EMT

### Genetically engineered mouse models (GEMMs)

Studies of PDAC is greatly accelerated by the availability of powerful GEMMs that recapitulates the oncogenic events and histologic progression of human PDAC. The most widely used KPC GEMM and their variants develop precancerous PanIN lesions at almost 100% penetrance at 8–10 weeks of age, and progress to developing locally advanced PDAC with dense desmoplasia at 16 weeks of age. The median survival of the KPC mice is around 5 months with the majority of mice developing malignant ascites and distant organ metastases.^47^ Notably, lineage-traced KPC model showed that a significant portion of PDAC cells have undergone EMT, evidenced by increased ZEB1 and decreased E-cadherin expression, and metastasized to the liver, in early PanIN stages.^17^ In contrary, using single-cell technology, PDAC cell population with mesenchymal signature was enriched in late, but not early stage PDAC tumors arising from *Ptf1a-Cre;INK4a*^*flox/flox*^*;LSL-KRAS*^*G12*^ (or KIC) and in *Pdx1-Cre;TP53*^*flox/flox*^*;LSL-KRAS*^*G12*^*(KPfC)* mice.^161^ Such discrepancy probably arises from the difference in definition of EMT in these two studies. It is critical to realize that EMT is a highly dynamic process that encompasses a wide spectrum of partial-EMT states.^10,12^ Therefore, changes in protein marker such as ZEB1 and E-cadherins protein expression, as described,^17^ may reflect a partial-EMT state where PDAC cells have not fully assumed the mesenchymal transcriptomic profile,^161^ but nonetheless are able to migrate and metastasize.

**Fig. 4:**
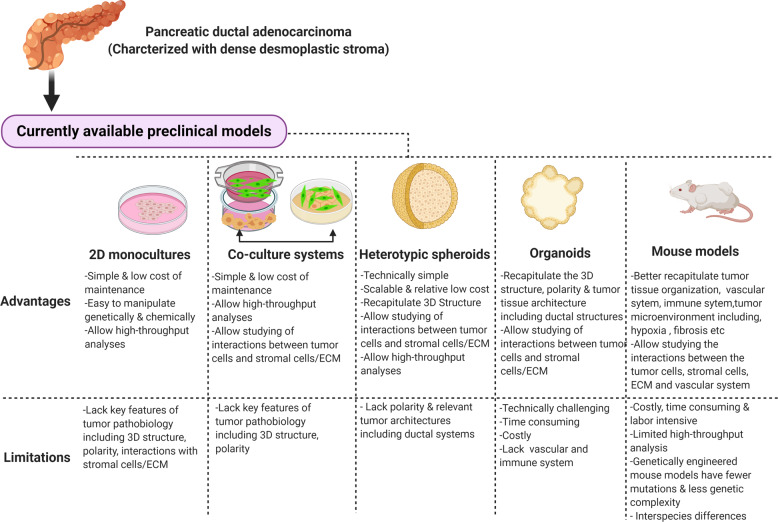
Summary of advantages and limitations of currently available preclinical models of PDAC. Desmoplastic stroma is a typical characteristic of pancreatic cancer tumor microenvironment that promote epithelial-to-mesenchymal transition (EMT) and therapeutic resistance. Improvement of in vitro models that include stromal cells including CAFs and immune cells should be increasingly incorporated to identify therapeutic strategies that can be further tested in mouse models

Importantly, the GEMMs also allow studies of intratumoral CAFs and immune cells during PDAC progression and will remain a powerful tool. Despite these advantages, the KPC models are costly, time-consuming, and labor intensive, limiting their use by most labs and for large scale drug or genetic screening. In addition, these GEMMs are typically driven by only few oncogenic events, typically deletion of a tumor suppressor and introduction of *KRAS*^*G12D*^, which do not reflect the genetic complexities of human PDAC. Therefore, in vitro human models that more closely recapitulate the heterotypic nature and biophysical environment of PDAC should be developed. As present stage, the traditional monolayer cell culture is still the most widely used method to study various aspects of PDAC biology, with considerable success in the pass. However, it is now increasingly clear that the behavior of PDAC cells and their response to genetic manipulation and therapeutic challenge is highly dependent on the culture condition and surrounding stromal cells.^162,163^ To this end, different heterotypic models to simulate native PDAC have been developed^163–166^ (Fig. 4).

### Heterotypic human cell co-culture models

A commonly adopted way to mimic the PDAC TME is by co-culturing differentially labelled, usually by fluorescence, PDAC cells and CAFs in monolayer cultures. These two cell types can be cultured with or without direct physical contact. For the former, PDAC cells and CAFs in fixed ratio are layered one on top of the other, or mingled, both of which allows bidirectional physical interactions. For the latter, these two cell types are housed in separate chambers (such as transwell systems) and cell–cell communication occurs only through diffusion of humoral factors. By mixed co-culture method, Ligorio et al. showed that secreted factors from CAFs can diversify PDAC into proliferative, EMT or double positive (DP) proliferative/EMT phenotypes, using single-cell RNAseq. The degree of diversification into these phenotypes is affected by the ratio of co-cultured CAFs. Mice injected with high-stroma mixture (90% CAFs:10% PDAC cells) developed more metastatic tumors that exhibit DP phenotype. Acquisition of EMT or DP phenotype is driven predominantly by TGF-β secreted from CAFs. In support, in human PDAC samples the DP phenotype is enriched in high-stroma tumors,^163^ whereas EMT phenotype is enriched in medium-stroma tumors. This study demonstrates that a relatively simple co-culture method, if conducted methodically and correlated carefully with human PDAC samples, can still faithfully recapitulate some aspects of real PDAC tumors. However, as recommended in the Banbury Center Consensus Statement,^44^ this relatively simple co-culture method can be further optimized by inclusion of matrices such as collagen, laminin, hyaluronan, as well as introduction of hypoxic and low serum conditions.

#### Spheroids

In actual tumors, PDAC cells are embedded three-dimensionally within the stroma. Following EMT, extravasated PDAC cells circulate within the blood stream as three-dimensional heterotypic clusters or single cells. Therefore, culturing PDAC cells in aggregates, or spheroids, as suspension is one-step closer to mimicking the real PDAC. In fact, basal oncogenic signaling and adaptive response to therapeutics are different when cancer cells are cultured in monolayer vs. three-dimentional (3D) spheroids.^167,168^ In addition, PDAC cells cultured as 3D spheroids produce more matrices, become more glycolytic and expresses more chemo-resistant genes compared to when grown as monolayers.^169^ PDAC cells with intact TGF-β signaling machinery assume more EMT phenotype when cultured as spheroids compared to monolayer culture.^170^ However, PDAC cells are also under constant interaction with surrounding cell types, and this can by mimicked by inclusion of CAFs, vascular endothelial cells and immune cells at various ratio to form heterotypic spheroids.^171^ Furthermore, addition of ECM matrices in the culture is commonly used to increase stiffness of the culture media. Several platforms such as Geltrex,^172,173^ biologically inert 3D alginate scaffolds^174^ and magnetic 3D bioprinting protocols^175^ have been developed to systematize spheroid generation and these are greatly helpful in high-throughput drug screens.

#### Organoids

In PDAC tumors, neoplastic ductal cells typically form single or multilayered epithelia with some degree of apical-basal polarity, forming dysfunctional ductal-like structures that are externally surrounded by basal membrane. During EMT, neoplastic cells further lose cellular polarity, breach the basement membrane, and invade into the vasculatures to metastasize. These are important cellular processes that cannot be recapitulated with monolayer or spheroid models. Culture method to isolate, grow, and propagate PDAC tumors with preserved cellular polarity and tissue architecture (termed organoids) were successfully developed by the Tuveson and Clevers groups using resected or biopsied human PDAC tissues.^176^ These PDAC organoids preserve the proteomic and transcriptomic features, and importantly their response to therapeutics that parallel their hosts in the clinic,^177^ making it currently the most powerful in vitro model with the closest proximity to primary PDAC. Moreover, single-cell transcriptomics showed that CAFs that were co-cultured with PDAC organoids, exhibited at least three different interchangeable subtypes: iCAFs, myCAFs, and apCAFs.^59,60^ However, establishment and expansion of PDAC organoids from biopsy samples typically takes weeks to months, require very specific cocktail of growth factors and Matrigel, and highly trained personnel to maintain the intratumoral heterogeneity. These caveats greatly limit the adoption of this technique in most research labs and must be overcome. Nonetheless, the organoid model has certainly gained traction in recent years for drug screening and personalized medicine.^178^

## Therapeutic targeting of PDAC stroma: recent lessons

It is widely accepted that PDAC stroma contributes to the poor outcome of PDAC ranging from EMT, metastasis, chemoresistance and immune evasion and is covered by a very nice review by Hosein et al.^2^ The major causes that underlie the poor prognosis of PDAC is extreme resistance to chemotherapy and early dissemination. In addition, PDAC has a much higher propensity to disseminate, compared to other cancer types such as lung and colon cancers that share similar oncogenic mutations. Lineage tracing in GEMMs showed that PDAC cells undergo EMT and metastasize even before formation of frank tumors, and these processes can be accelerated by inflammation.^17^ Similarly, circulating tumor cells are detected in most PDAC patients at any stage.^179^ A nice review by Kalluri and Weinberg proposed three different types of EMT.^180^ Type II EMT was precipitated by chronic inflammation and fibrosis, which (1) provide humoral factors that promote EMT, and (2) disrupt tissue architecture thereby allowing EMT cells to escape. Therefore, chronic inflammation and the associated fibrosis contribute to the aggressive nature of PDAC starting from early stage. However, strategies that targeting the stroma component of PDAC have unfortunately failed in large scale clinical trials. In particular, addition of pegylated hyaluronidase (PEGPH20) to chemotherapy (FOLFIRINOX or Gemcitabine/Abraxane) failed to improve patient survival in large scale clinical studies,^181,182^ despite promising results in GEMMs.^117^ Although disappointing, these studies also provided precious lessons for future improvement. First, the content of PDAC ECM is highly dynamic and consists of several matrix proteins that may compensate for reduced hyaluronan abundance. Second, hyaluronan is a rapidly turned over proteoglycan, synthesized by three different hyaluronan synthases (HAS1-3). It is possible that in clinical trials the administered PEGH20 dose, hindered by side effects including gastrointestinal toxicities and thromboembolic events when combined with chemotherapy, was inadequate to outpace hyaluronan synthesis. Third, degradation of hyaluronan may results in a surplus of low and medium molecular weight hyaluronan species that still activate CD44 and induce protective EMT. Fourth, the overall utility of KPC GEMMs in predicting clinical response should be carefully reconsidered, especially since several promising therapeutic strategies published based on these models have so far failed to translate into clinical success. While the KPC GEMMs have unquestionably contributed enormously to the understanding of PDAC biology that cannot otherwise be achieved with conventional cell lines, patient-derived xenografts or other in vitro models, it is equally important to improve human cell-based models using various strategies to complement knowledge derived from GEMMs. Fifth, therapeutic strategies that target critical signaling pathways in both PDAC and CAFs should be developed. For example, inhibition of focal adhesion kinase (FAK) markedly reduces stromal collagen and also sensitizes PDAC cells to chemotherapy.^108^ Targeting the NF-κB pathway through suppressing Interleukin-1 receptor associated kinase 4 (IRAK4) sensitizes PDAC cells to genotoxic stress and simultaneously lowers the ability of CAFs to deposit collagen and foster tumor fibrosis, leading to improved response to chemotherapy.^54,92,183^

## Conclusions and prospects

PDAC is currently the only major cancer type that has not benefited from targeted or immune-based therapies, despite decades of research and extensive publications. Recent modest improvement in survival of PDAC patients results almost exclusively from improvement in chemotherapy and perioperative care. An important factor that must be critically re-examined is the methodologies that are widely used to studying this histologically complicated cancer type. The dense stroma of PDAC plays a very active role in modulating the behavior and therapeutic response of PDAC cells, and induction of EMT as we reviewed here, is merely one of many recalcitrant facets of PDAC. Therefore, incorporation on stromal elements including CAFs, ECM and immune cells and proper culture conditions including hypoxia and low serum, should be increasingly adopted in future studies of any signaling pathway in PDAC. A robust, 3D heterotypic human cell-based culture model should also be established for unbiased high-throughput screens and drug discovery. Importantly, the experimental models should be cost-effective, standardized and reproducible, all of which require extensive discussion and establishment of consensus or guidelines by experts in the field, as has been done for CAF^44^ and EMT^12^ research. To increase the chance of future success, any novel therapeutic regimens should be rigorously tested and shown to be effective in both heterotypic human culture models and GEMMs before being advanced into clinical trials.
